# Attitudes and practices of Chinese physicians regarding chronic kidney disease and acute kidney injury management: a questionnaire-based cross-sectional survey in secondary and tertiary hospitals

**DOI:** 10.1007/s11255-018-1882-1

**Published:** 2018-05-10

**Authors:** Yanhua Wu, Yuanhan Chen, Shixin Chen, Yani He, Huaban Liang, Wei Dong, Xinling Liang

**Affiliations:** 1 Division of nephrology, Guangdong General Hospital, Guangdong Academy of Medical Sciences, 106, Zhong Shan Road 2, Guangzhou, 510080 Guangdong China; 2 Division of Preventive Medicine, School of Public Health, Guangzhou Medical University, 95, Dongfeng West Road, Guangzhou, 510182 Guangdong China; 3 Division of Nephrology, Daping Hospital, Research Institute of Surgery, Army Medical University, Chongqing, 400042 China

**Keywords:** Chronic kidney disease, Acute kidney injury, Surveys and questionnaires, Disease management, Internet

## Abstract

**Objective:**

This questionnaire-based cross-sectional survey reported the attitudes and practices of Chinese doctors regarding chronic kidney disease (CKD) and acute kidney injury (AKI) management.

**Methods:**

An online questionnaire consisting of general information, awareness of CKD and AKI, education status, renal laboratory items, and clinical practices between February 20, 2017 and August 15, 2017.

**Results:**

Among the 1289 respondents from secondary and tertiary hospitals in 30 provinces, 718 (55.7%) were nephrologists, 94.3% had the ability to evaluate glomerular filtration rates, and 98.8% could evaluate urinary protein excretion, indicating that Chinese doctors met the minimum requirements to manage CKD. However, nearly half of all respondents reported that easy methods for spot urine creatinine-adjusted urinary protein assessments were unavailable. Awareness of the CKD risk stratification system and AKI definition was inadequate, and only 54.2% of respondents reported that they had received nutritional education for renal diseases. Although most of the respondents were nephrologists at university hospitals, 66.4% and 76.3% of respondents reported nephrology referrals and nephrology consultations, respectively, after AKI, suggesting an insufficient role for nephrologists. Finally, management models differed significantly, indicating that universal guidelines for CKD and AKI management are required across China.

**Conclusions:**

Several considerable challenges remain regarding CKD and AKI management in China, including inadequate knowledge and training systems, an absence of clinical protocols, and insufficient multidisciplinary cooperation.

**Electronic supplementary material:**

The online version of this article (10.1007/s11255-018-1882-1) contains supplementary material, which is available to authorized users.

## Introduction

Chronic kidney disease (CKD) and acute kidney injury (AKI) are two interconnected kidney diseases [1–3]. The kidneys are not only target organs of many diseases but can also strikingly aggravate or initiate systemic pathophysiological processes through their complex functions and effects on total body homoeostasis. Increasing evidence suggests that CKD and AKI not only contribute to end-stage renal disease but are also associated with severe extra-renal morbidities and long-term mortality [4–6].

Two CKD guidelines have been introduced in China, the Kidney Disease Outcomes Quality Initiative (K/DOQI) Guideline from the National Kidney Foundation of America [7] and the Kidney Disease: Improving Global Outcomes (KDIGO) 2012 Guideline from the International Society of Nephrology [8]. Glomerular filtration rate (GFR) is the most important criteria for classifying CKD and is measured by radioisotopic methods, which is the gold standard in China, or estimated through a mathematical formula using the results of serum creatinine (Scr), cystatin C (Cys C), or both. Estimated GFR (eGFR) is often confused with the previously widely used creatinine clearance rate (CCr). The units of CCr are mL/min, while the units of GFR or eGFR are mL/min/1.73 m^2^. In recent years, proteinuria has also been recognized as a paradigm for the risk stratification of CKD [9–11], and was adopted by the 2012 KDIGO guideline [8]. There are four ways to evaluate urinary protein excretion: dipstick test, protein-to-creatinine ratio (PCR) or albumin-to-creatinine ratio (ACR) in spot urine, and 24-h urine protein quantity. Because urine dipstick tests in spot urine are inaccurate, the other three are recommended.

The AKI definition was first derived from contrast media-induced nephrotoxicity, also called contrast-induced AKI (CI-AKI), which was proposed by the European Society of Urogenital Radiology in 1999. Afterwards, three AKI criteria were successively introduced in China [12]: the risk, injury, failure, loss, and end-stage (RIFLE) for renal diseases, the acute kidney injury network (AKIN) classification systems, and the KDIGO clinical consensus. AKI is defined and classified by the percentage increases in Scr or Cys C or decrease in urine output rate, if available.

Poor general knowledge and low medical resources might limit CKD and AKI management in developing countries [1, 2, 13]. China is a developing country of more than 1.3 billion people that is rapidly transitioning into a developed economy, which has been accompanied by changes in China’s health care system [14]. A recent series of epidemiological studies have reported that China is a country with a high prevalence of CKD and AKI [15–17]. These large data reports depicted characteristics from the patient’s perspective; however, CKD and AKI management from a physician’s viewpoint was not investigated. Because of the serious shortage of dietitians in China, doctors play a multifunctional role for kidney disease, including diagnosis, treatment, patient education, and dietary management. In this questionnaire-based cross-sectional survey, we reported attitudes and practices of > 1000 Chinese doctors regarding CKD and AKI management.

## Methods

A survey including general information of the investigated hospitals and doctors, awareness of CKD and AKI, education status, renal laboratory items, and clinical practices was conducted on an internet questionnaire survey platform (https://www.wjx.cn/) in the form of an electronic questionnaire (attached in the supplementary data 1) with 42 multiple choice questions and one open-ended question (city where the respondent worked). Because the questions were related to the ability of hospitals and doctors, names of hospitals and doctors were not collected for privacy protection.

The questionnaire was distributed between February 20, 2017 and August 15, 2017 through medical social media attached with a statement that it was only for doctors. And nephrology doctors at the South China Forum on blood purification (July 2017) were voluntarily invited to join the survey. Moreover, the question “How long have you been a doctor?” was Question 4 of Part I (supplementary data 1). Thus, we regarded all the respondents as doctors. The protocol for this study was approved by the ethics committee of Guangdong General Hospital (GDREC.2016327H). To ensure response reliability, questionnaires completed in < 3 min were excluded. For self-evaluations of knowledge about CKD and AKI, questioned doctors could read sample answers that followed the question.

Data are expressed as the percentage of positive answers to each question. To describe differences among hospitals at different levels, respondents were further subclassified into secondary or tertiary hospitals and university or non-university hospitals.

## Results

### Characteristics of surveyed doctors

We received 1446 completed questionnaires. Because doctors in secondary and tertiary hospitals are the main users of CKD and AKI management, some questionnaires from primary hospitals were not included in the final analysis. Among the responses, 157 were excluded, 64 because of omitting hospital level, 77 for short completion times, and 16 for being from primary hospitals.

Among the included 1289 respondents, 617 (47.9%) were male, the median age was 36 years (range 30–60 years), and 718 (55.7%) were nephrologists; the majority of responses were from senior doctors. The number of respondents with 5–10, 10–20, 20–30, and > 30 years of professional experience was 287 (22.3%), 472 (36.6%), 272 (21.1%), and 76 (5.9%), respectively. The number of respondents with bachelors, masters, and doctorate degrees was 720 (55.9%), 388 (30.1%), and 116 (9.0%), respectively. The respondents were from hospitals in 30 provinces, and approximately one-third were from Guangdong Province (Supplementary data 2). Regarding hospital settings, 97.3% of the doctors were from hospitals with hemodialysis centers, 348 (27.0%) and 941 (73.0%) were from secondary and tertiary hospitals, respectively, and 788 (61.1%) were from Class A tertiary hospitals. Among the 1045 doctors from university hospitals, 392 (30.4%) and 653 (50.7%) were from affiliated and non-subordinate affiliated hospitals, respectively.

### Knowledge of CKD and AKI

**Fig. 1:**
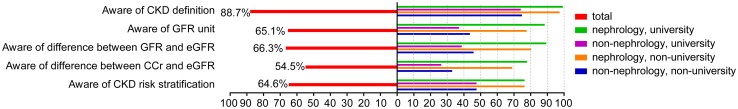
Percentage of doctors with CKD awareness. *CKD* chronic kidney disease, *GFR* glomerular filtration rate, *CCr* creatinine clearance rate, *eGFR* estimated GFR

Almost all nephrology doctors reported that they knew the CKD definition regardless of whether they were from a university hospital or not; however, a quarter of non-nephrology doctors did not (Fig. 1). Despite this high percentages of CKD awareness, a similarly high percentage of GFR unit awareness was not reported, nor was there awareness of the differences among CCr, GFR, and eGFR (Fig. 1). Additionally, 35.4% of the respondents reported that they did not know the GFR and albuminuria risk stratification system, including more than 20% of nephrologists and more than half of the non-nephrology doctors (Fig. 1). These discrepancies suggested that awareness of the CKD definition might be significantly overestimated. In accordance with the limited awareness of CKD details, only 46.9% of the doctors reported that they had adopted the K/DOQI and/or KDIGO guidelines in their clinical practice.

**Fig. 2:**
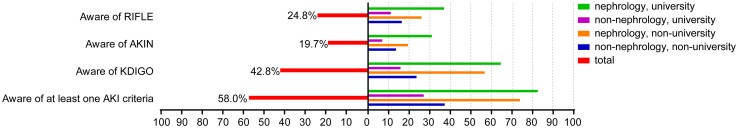
Percentage of doctors with AKI awareness. *AKI* acute kidney injury, *RIFLE* risk, injury, failure, loss, and end-stage renal disease, *AKIN* acute kidney injury network, *KDIGO* Kidney Disease: Improving Global Outcomes

In total, 58.0% of respondents reported that they were aware of at least one AKI criteria. Among the three AKI criteria, the KDIGO criteria was the most well known, with a total awareness percentage of 42.8%, despite it being the most recently published. The KDIGO awareness percentage was more than 50% lower among non-nephrology doctors compared with nephrologists (Fig. 2), indicating the importance of nephrologists in AKI management.

### Education status

**Fig. 3:**
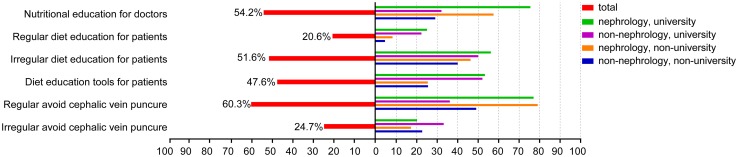
Percentages of doctors and patients who received nephrology education

Only 54.2% of respondents reported that they had received nutritional education for renal diseases, and trained doctors were mostly nephrologists from university hospitals. Only 20.6% of doctors reported that they gave regular dietary lectures to CKD patients, and 51.6% reported that dietary education was carried out irregularly (Fig. 3). This indicated that patients in the remaining 28.8% of respondents’ hospitals were not educated, including some university hospitals.

Avoiding cephalic vein puncture is obligatory for the prophylactic protection of vascular access in advanced-stage CKD. Nearly 80% of nephrologists observed this guideline, and the remainder practiced it irregularly. However, more than half of the non-nephrologists did not adhere to this rule (Fig. 3). Considering that almost all of the respondents were from hospitals with hemodialysis centers, this percentage of prophylactically protecting vascular access requires further improvements.

### Clinical test items for nephrology examination

**Fig. 4:**
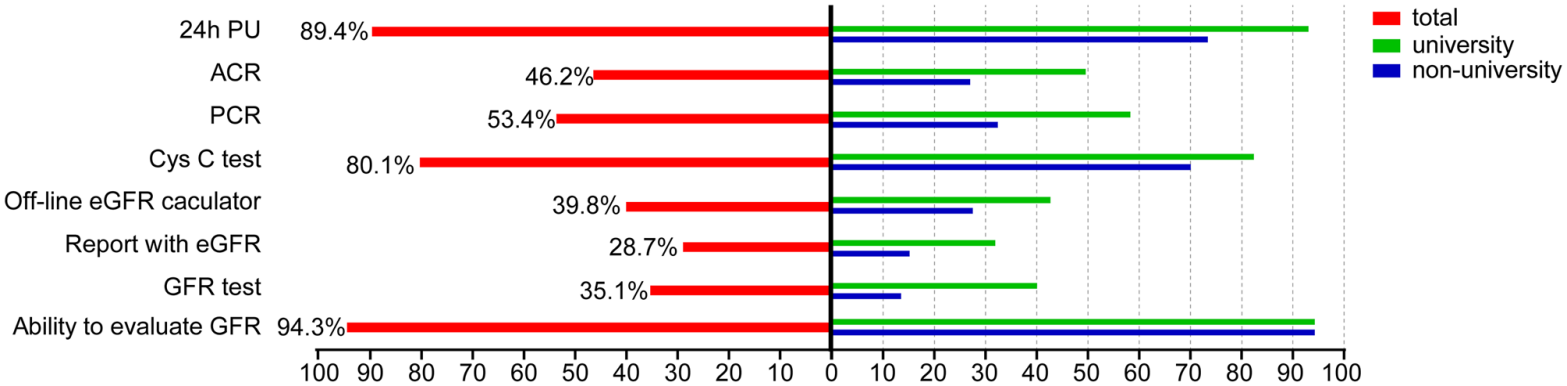
Percentage of medical facilities containing renal laboratory items. *PU* proteinuria, *ACR* albumin-to-creatinine ratio, *PCR* protein-to-creatinine ratio, *Cys C* cystatin C, *GFR* glomerular filtration rate, *eGFR* estimated GFR

Among the 1179 respondents, 1165 (98.8%) reported that they could evaluate urinary protein excretion using any of the three recommended methods; however, only 46.2 and 53.4% of respondents reported that ACR and PCR in spot urine was available, respectively. Among the 110 doctors who did not respond to this question, 56 (50.9%) were nephrologists (Fig. 4). The reason for the lack of responding was speculated to be that they did not fully understand these methods.

More than 70% of respondents reported that Cys C measurements were available at their hospitals (Fig. 4). Radioisotopic methods for evaluating GFR were reported for 35.1% of respondents’ hospitals. Besides this nuclear medicine procedure, 39.8% of the doctors reported that they used medical software to calculate eGFR, and 28.7% reported that an automatically calculated eGFR value was provided with creatinine values in reports from their hospital. In total, 94.3% of doctors could evaluate GFR (Fig. 4).

### Practices for renal disease management

**Fig. 5:**
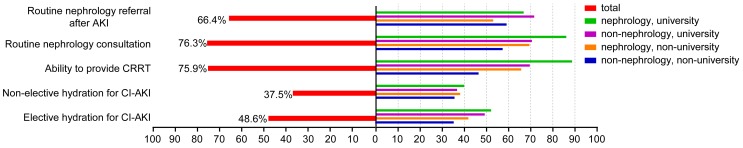
Practices for renal disease management. *AKI* acute kidney injury, *CRRT* continuous renal replacement therapy, *CI-AKI* contrast-induced AKI

We first investigated the current status of multidisciplinary cooperation. In total, 66.4% of respondents reported nephrology referrals and 76.3% reported nephrology consultations for AKI (Fig. 5). However, because the percentage of AKI misdiagnoses is very high in China [18], these relatively high percentages of nephrology care following AKI might be overestimated. Then, continuous renal replacement therapy (CRRT) was further surveyed to evaluate the ability to treat critical AKIs. Nearly a quarter of doctors reported that their hospitals had no ability to provide CRRT, which was significant in non-university hospitals. Finally, prophylactic hydration to protect CI-AKI before contrast media exposure was inquired to evaluate the strategy for this type of preventable AKI. In total, 13.9% of doctors reported a non-prophylactic hydration strategy, while 48.6% reported a non-elective hydration strategy regardless of risk stratification (Fig. 5).

## Discussion

This is the first study to survey the current attitudes and practices of CKD and AKI management from the perspective of Chinese doctors. Our results showed that almost all doctors reported that they could evaluate urinary protein excretion and GFR (or eGFR), which suggested that Chinese doctors met the minimum requirement to evaluate the risk stratification of these two renal diseases. However, the following barriers should be accounted for in the future.

First, awareness of CKD risk stratification systems and the AKI definition were inadequate. According to the 2016 Statistic Bulletin on Health Development, medical personnel who had either bachelor or post-bachelor degrees accounted for 32.3% of all healthcare workers in China. However, doctors from the lowest level hospitals were excluded from this study, and 95% of surveyed doctors had bachelor or post-bachelor degrees. Thus, it is speculated that the actual level of awareness is more serious than what our report showed.

Second, a lack of training is an urgent burden for Chinese Continuing Medical Education. A nationwide survey indicated that only 8000 doctors were registered as nephrologists in 2008, which is equivalent to one nephrologist per 15,000 CKD patients or one nephrologist per 360 hospitalized AKI patients [12, 16]. In addition to this dearth of nephrologists, many who are currently practicing are not well-trained, such as in specialized dietary knowledge, which results in insufficiently educated patients.

Third, CKD and AKI management significantly varied among hospitals. The more convenient methods (ACR and PCR) for evaluating proteinuria are not routine assays, which could limit CKD risk evaluation, especially in outpatient setting. Further, many hospitals cannot yet perform CRRT. The Chinese government is trying to construct a medical referral system to transfer critical patients from local hospitals to higher level hospitals. However, before this system is active, how to treat patients who need timely CRRT at local hospitals remains a considerable problem. Additionally, opinions differ greatly on the efficacy of prophylactic hydration in preventing CI-AKI, and these diverse opinions drive the use or lack of use of hydration in Chinese patients. Even in a randomized controlled trial for CI-AKI in China, half of the patients received prophylactic hydration while the other half did not [19]. This heterogeneity might be attributed to the fact that CKD and AKI management in China is mostly empirical, and few randomized controlled trials have been conducted. For example, the recent AMACING trail questioned the need of prophylactic hydration for CI-AKI by weighting non-significant effect versus additional medical costs [20]. In the Dutch AMACING trail, mean in-hospital costs were about 120–230 Euros higher in patients who received prophylactic hydration compared with non-hydrated patients. However, the medical cost associated with hydration is almost negligible in China because of the medical price system. Thus, a practice model suitable for nephrology management is essential in China.

Finally, nephrology referrals and consultations were not routine practice in many hospitals. Early nephrology referrals are associated with better renal prognoses and lower in-hospital mortality rates [21]. In contract, the inadequate role of nephrologists might exacerbate renal and extra-renal prognoses.

This survey was conducted in a non-randomized way through social media, which may have led to selection bias. Additionally, several limitations are associated with questionnaire-based surveys. Awareness percentages might be overestimated when respondents are aware of only part of the questions. To reduce this fallibility, we provided answers or relevant information to help them give an actual evaluation. We also examined survey details to confirm whether the respondents fully understood the questions. For example, when we queried CKD awareness, we tested not only the CKD definition but also the eGFR units. We believe that this will also serve as a training process for respondents who did not have a mastery of nephrology. Furthermore, because hospitals and doctors were anonymous, responses from doctors at the same hospital could not be discriminated; thus, we could not perform a homogeneity analysis. However, anonymity improved reliability. Finally, the reported questionnaire results were subjective and could not be used to truly identify what is happening in real world situations.

In conclusion, most Chinese doctors met the minimum requirements for CKD and AKI management. However, several considerable challenges remain, including inadequate knowledge and training systems, the absence of clinical protocols and an immature multidisciplinary system.

## Electronic supplementary material

Below is the link to the electronic supplementary material.

Supplementary material 1 (DOCX 23 KB)

Supplementary material 2 (DOCX 13 KB)
