# Supplementary material for: Attitudes and practices of Chinese physicians regarding chronic kidney disease and acute kidney injury management: a questionnaire-based cross-sectional survey in secondary and tertiary hospitals
Source: Int Urol Nephrol. 2018 May 10;50(11):2037–42. doi: 10.1007/s11255-018-1882-1 (PMC6208760; doi:10.1007/s11255-018-1882-1)
Supplement: Supplementary file 1 — Supplementary material 1 (DOCX 23 KB) [file 11255_2018_1882_MOESM1_ESM.docx]

**Supplementary data 1**

**A Questionnaire On Chronic Kidney Disease And Acute Kidney Injury In Chinese Hospitals**

Some questions have brief answers; you may answer the questions after reading the sample responses below, but we would prefer for you to submit your answers prior reading those provided.

**Part I: Basic information**

1. Your age is: _______ years.
2. Your gender is:
3. Male
4. Female
5. Your highest degree is:
   1. Junior college
   2. Undergraduate
   3. Masters
   4. Doctorate and above
6. How long have you been a doctor? _______ year(s).
7. How long have you been a nephrologist*? _______ year(s).

**Non-nephrologists, please choose 0 years.*

1. Your hospital is located in: ______________ (Province), ______________ (City).
2. The level of your hospital is: Class: ______________, Grade: ______________.
3. Your hospital belongs to:
   1. Public
   2. Private
4. Your hospital type is:
   1. Non-teaching hospital
   2. Teaching hospital directly under a medical university
   3. Teaching hospital indirectly under a medical university
5. Does your hospital provide hemodialysis treatment?
   1. Yes
   2. No
6. Does your hospital provide peritoneal dialysis?
7. Yes
8. No
9. Does your hospital provide bedside hemodialysis?
10. No
11. Nephrology department to manage
12. Intensive care unit to manage
13. Does your hospital provide continuous renal replacement therapy?
14. Yes
15. No

**Part II:** **Degree of understanding chronic kidney disease (CKD) among respondents**

1. Do you know the exact definition of chronic kidney disease (CKD)?
2. Yes
3. No
4. Do you understand and apply any chronic renal disease guidelines?
5. Do not understand or apply.
6. Understand the K/DOQI guidelines, but do not treat CKD according to them.
7. Understand the K/DOQI guidelines, and treat CKD according to them.
8. Understand the KDIGO guidelines, but do not treat CKD according to them.
9. Understand the KDIGO guidelines, and treat CKD according to them.
10. Understand the K/DOQI and KDIGO guidelines, and treat CKD according to both.
11. Other Situations: _________________________________________________________.
12. Do you understand GFR and proteinuria risk stratifications?
13. Yes
14. No
15. Do you know the difference between GFR and eGFR*?
16. Yes
17. No

**If* ***Yes****, proceed to the next question; if* ***No****, jump to Part III.*

1. Do you know the units of GFR?
2. Yes
3. No
4. Do you know the primary difference between endogenous creatinine clearance and eGFR?

a) Yes

b) No

**Part III: Degree of understanding acute kidney injury (AKI) among respondents**

1. Which are you more likely to encounter in your practice?
2. Acute renal failure
3. Acute kidney injury
4. Do you know the RIFLE/AKIN/KDIGO classification criterions for AKI? (select all that apply)
5. None
6. RIFLE classification criteria for AKI
7. AKIN classification criteria for AKI
8. KDIGO classification criteria for AKI

**Part IV: Conditions of kidney disease diagnosis and treatment**

1. Are the following laboratory tests performed in your hospital? (select all that apply)
2. Serum cystatin C
3. Nuclide examination of glomerular filtration rate

**If* ***Yes****, continue to choose:*

- 1. When adopting surface area corrections, are the units of the result: ml/(min×1.73 m^2^)?
  2. Yes
  3. No

1. Quantitative examination of urinary protein

**If* ***Yes****, continue to choose:*

- 1. Laboratory testing includes: (select all that apply)
     1. 24 h urine protein
     2. Urine protein creatinine ratio
     3. Urine albumin creatinine ratio

1. Random urine protein concentration (mg/dL) without creatinine correction
2. The units of urine protein tests in your hospital include: (select all that apply)
3. mg/g
4. mg/L
5. mg/mol
6. mg/mmol
7. Is eGFR automatically calculated in creatinine inspection reports?
8. Yes
9. No

**Part V: Practices of kidney disease diagnosis and treatment**

1. Do you know the eGFR formula (you do not need to remember the coefficients exactly)? (select all that apply)
2. No
3. Understand the EPI formula
4. Understand the MDRD formula
5. Other situations: _________________________________________________________.
6. Is there computer software available at your hospital to calculate eGFR?
7. No
8. Yes
   1. If **Yes**, do you use it?
      1. Yes
      2. No
   2. If **No**, would you like to get such software?
      1. Yes*
      2. No

**If* ***Yes****, please leave your e-mail address or WeChat ID: ____________________.*

1. Do you have mobile phone software or a network eGFR calculator?
2. No
3. Yes
   1. If **Yes**, do you use it?
      1. Yes
      2. No
   2. If **No**, would you like to get such software?
      1. Yes*
      2. No

**If* ***Yes****, please leave your e-mail address or WeChat ID: ____________________.*

1. For a patient with eGFR or GFR of 55 ml/min/1.73 m^2^, would you diagnose chronic renal disease (CKD)?
2. Never
3. Sometimes
4. Normally
5. If **Never**, why would you “never” diagnose this patient with CKD?
   1. Do not understand CKD
   2. Do not know how to calculate eGFR
   3. No ICD coding for CKD
6. Have you ever received nutritional training for CKD?
   1. Yes
   2. No
7. Are there any educational props to assist teaching CKD dietary information in your hospital?
   1. Yes
   2. No
8. Are there educational programs that teach dietary information to CKD patients at your hospital?
   1. Never
   2. Unscheduled
   3. Scheduled
9. Would you advise non-dialysis stage 4–5 CKD patients to avoid left forearm vein puncture?
10. Never
11. Sometimes
12. Normally
13. Is there any acute kidney injury-related ICD coding in your hospital?
14. Unsure
15. Yes
16. No
17. Do patients at your hospital regularly receive hydration treatment before angiography or enhanced CT?
    - 1. Never
      2. Some high-risk patients
      3. Normally
18. Your department is:
    1. Nephrology
    2. Non-nephrology
       1. If **Nephrology**, do other departments require a nephrology consult for CKD or AKI?
19. Never
20. Scarcely
21. Occasionally
22. Normally
23. If **Non-nephrology**, do you require a nephrology consult for CKD or AKI.
24. Never
25. Scarcely
26. Occasionally
27. Normally
28. Other situations: __________________________________________________.
29. According to your experiences, do AKI patients need to be admitted to nephrology follow-up after hospital discharge?
    1. Never
    2. Scarcely
    3. Occasionally
    4. Normally

Other situations: ________________________________________________
