# Supplementary material for: Attitudes and practices of Chinese physicians regarding chronic kidney disease and acute kidney injury management: a questionnaire-based cross-sectional survey in secondary and tertiary hospitals
Source: Int Urol Nephrol. 2018 May 10;50(11):2037–42. doi: 10.1007/s11255-018-1882-1 (PMC6208760; doi:10.1007/s11255-018-1882-1)
Supplement: Supplementary file 2 — Supplementary material 2 (DOCX 13 KB) [file 11255_2018_1882_MOESM2_ESM.docx]

| **Province** | **Doctors Number** |
| --- | --- |
| Anhui | 5 |
| Beijing | 1 |
| Fujian | 22 |
| Gansu | 15 |
| Guangdong | 459 |
| Guangxi | 29 |
| Guizhou | 9 |
| Hainan | 11 |
| Hebei | 3 |
| Henan | 19 |
| Hubei | 50 |
| Hunan | 64 |
| Jilin | 80 |
| Jiangsu | 8 |
| Jiangxi | 89 |
| Liaoning | 1 |
| Neimenggu | 100 |
| Ningxia | 1 |
| Qinghai | 15 |
| Shandong | 3 |
| Shanxi (capital city is Taiyuan) | 1 |
| Shanxi (capital city is Xi’an) | 96 |
| Shanghai | 26 |
| Sichuan | 9 |
| Tianjin | 1 |
| Xizang | 4 |
| Xingjiang | 35 |
| Yunnan | 16 |
| Zhejiang | 70 |
| Chongqing | 47 |
| Total | 1289 |
